# Spatiotemporal transmission of SARS-CoV-2 lineages during 2020–2021 in Pernambuco—Brazil

**DOI:** 10.1128/spectrum.04218-23

**Published:** 2024-04-23

**Authors:** Lais Ceschini Machado, Filipe Zimmer Dezordi, Gustavo Barbosa de Lima, Raul Emídio de Lima, Lilian Caroliny Amorim Silva, Leandro de Mattos Pereira, Alexandre Freitas da Silva, Antonio Marinho da Silva Neto, André Luiz Sá de Oliveira, Anderson da Costa Armstrong, Rômulo Pessoa-e-Silva, Rodrigo Moraes Loyo, Barbara de Oliveira Silva, Anderson Rodrigues de Almeida, Maira Galdino da Rocha Pitta, Francisco de Assis da Silva Santos, Marilda Mendonça Siqueira, Paola Cristina Resende, Edson Delatorre, Felipe Gomes Naveca, Fabio Miyajima, Tiago Gräf, Rodrigo Feliciano do Carmo, Michelly Cristiny Pereira, Tulio de Lima Campos, Matheus Filgueira Bezerra, Marcelo Henrique Santos Paiva, Gabriel da Luz Wallau, On behalf of Fiocruz COVID-19 Genomic Network

**Affiliations:** 1 Departamento de Entomologia, Instituto Aggeu Magalhães (IAM)-Fundação Oswaldo Cruz-FIOCRUZ, Recife, Pernambuco, Brazil; 2 Núcleo de Bioinformática (NBI), Instituto Aggeu Magalhães (IAM), FIOCRUZ-Pernambuco, Recife, Pernambuco, Brazil; 3 Núcleo de Plataformas Tecnológicas (NPT), Instituto Aggeu Magalhães (IAM), FIOCRUZ-Pernambuco, Recife, Pernambuco, Brazil; 4 Núcleo de Estatística e Geoprocessamento, Instituto Aggeu Magalhães (IAM)- Fundação Oswaldo Cruz Pernambuco- FIOCRUZ-PE, Recife, Brazil; 5 Colegiado de Medicina, Universidade Federal do Vale do São Francisco, Petrolina, Brazil; 6 Suely-Galdino Therapeutic Innovation Research Center (NUPIT-SG), Federal University of Pernambuco (UFPE), Recife, Pernambuco, Brazil; 7 Departamento de Parasitologia, Instituto Aggeu Magalhães (IAM), FIOCRUZ-Pernambuco, Recife, Pernambuco, Brazil; 8 Núcleo de Ciências da Vida, Universidade Federal de Pernambuco (UFPE), Centro Acadêmico do Agreste, Caruaru, Brazil; 9 Laboratory of Respiratory Viruses and Measles (LVRS), Instituto Oswaldo Cruz, FIOCRUZ-Rio de Janeiro, Rio de Janeiro, Brazil; 10 Departamento de Biologia, Centro de Ciências Exatas, Naturais e da Saúde, Universidade Federal do Espírito Santo, Alegre, Espírito Santo, Brazil; 11 Laboratório de Ecologia de Doenças Transmissíveis na Amazônia (EDTA), Instituto Leônidas e Maria Deane, FIOCRUZ-Amazonas, Manaus, Amazonas, Brazil; 12 Analytical Competence Molecular Epidemiology Laboratory (ACME), FIOCRUZ-Ceará, Fortaleza, Ceará, Brazil; 13 Laboratório de Virologia Molecular, Instituto Carlos Chagas, Fundação Oswaldo Cruz, Curitiba, Paraná, Brazil; 14 Colegiado de Ciências Farmacêuticas, Universidade Federal do Vale do São Francisco, Petrolina, Brazil; 15 Departamento de Microbiologia, Instituto Aggeu Magalhães (IAM), FIOCRUZ-Pernambuco, Recife, Pernambuco, Brazil; 16 Department of Arbovirology, Bernhard Nocht Institute for Tropical Medicine, WHO Collaborating Center for Arbovirus and Hemorrhagic Fever Reference and Research, National Reference Center for Tropical Infectious Diseases, Hamburg, Germany; Uniwersytet Medyczny w Bialymstoku, Bialystok, Poland

**Keywords:** genomic surveillance, COVID-19, lineage replacement, state level

## Abstract

SARS-CoV-2 virus emerged as a new threat to humans and spread around the world, leaving a large death toll. As of January 2023, Brazil is among the countries with the highest number of registered deaths. Nonpharmacological and pharmacological interventions have been heterogeneously implemented in the country, which, associated with large socioeconomic differences between the country regions, has led to distinct virus spread dynamics. Here, we investigate the spatiotemporal dispersion of SARS-CoV-2 lineages in the Pernambuco state (Northeast Brazil) throughout the distinct epidemiological scenarios that unfolded in the first 2 years of the pandemic. We generated a total of 1,389 new SARS-CoV-2 genomes from June 2020 to August 2021. This sampling captured the arrival, communitary transmission, and the circulation of the B1.1, B.1.1.28, and B.1.1.33 lineages; the emergence of the former variant of interest P.2; and the emergence and fast replacement of all previous variants by the more transmissible variant of concern P.1 (Gamma). Based on the incidence and lineage spread pattern, we observed an East-to-West to inner state pattern of transmission, which is in agreement with the transmission of more populous metropolitan areas to medium- and small-size country-side cities in the state. Such transmission patterns may be partially explained by the main routes of traffic across municipalities in the state. Our results highlight that the fine-grained intrastate analysis of lineages and incidence spread can provide actionable insights for planning future nonpharmacological intervention for air-borne transmissible human pathogens.

**IMPORTANCE:**

During the COVID-19 pandemic, Brazil was one of the most affected countries, mainly due its continental-size, socioeconomic differences among regions, and heterogeneous implementation of intervention methods. In order to investigate SARS-CoV-2 dynamics in the state of Pernambuco, we conducted a spatiotemporal dispersion study, covering the period from June 2020 to August 2021, to comprehend the dynamics of viral transmission during the first 2 years of the pandemic. Throughout this study, we were able to track three significant epidemiological waves of transmission caused by B1.1, B.1.1.28, B.1.1.33, P.2, and P.1 lineages. These analyses provided valuable insights into the evolution of the epidemiological landscape, contributing to a deeper understanding of the dynamics of virus transmission during the early years of the pandemic in the state of Pernambuco.

## INTRODUCTION

Sustained SARS-CoV-2 human transmission was first characterized as an unknown/unusual pneumonia in the province of Wuhan in China in December 2019 (1). Since then, this virus has spread worldwide, causing one of the largest pandemics recorded by a respiratory virus. To date (November 2022), up to 628 million diagnosed cases are known and more than 6 million deaths were registered worldwide (2). Due to recent technological and scientific advances in genome sequencing and bioinformatic analysis, it has been possible to closely monitor SARS-CoV-2 evolution in symptomatic patients and detect variants of concern (VOCs) and interest (VOIs) (3). VOCs carry a combination of spike amino acid changes and indels that enhance receptor binding to human ACE2 receptors and/or immune escape capacity from specific neutralizing antibodies (4–6), besides many other mutations along the genome that are poorly studied so far (7).

The world experienced the emergence of the first generation of VOCs by the end of 2020, such as Alpha (B.1.1.7), Beta (B.1.351), and Gamma (P.1), which variably spread among countries, but all caused massive surges in cases mostly in the countries where they were first detected (8–10). It was then followed by a second generation of VOCs, such as Delta and Omicron, which were more transmissible and carried a large array of immune escape mutations (9–11). Both lineages are substantially more transmissible than previous circulating lineages and rapidly replaced them (11–14). For the Delta lineages, there was a direct association between increasing frequency and sustained increment in diagnosed cases in European countries, while in Brazil, Delta replacement occurred during a sustained decrease in cases (15, 16). On the other hand, Omicron has shown a unique immune escape profile and has spread globally, replacing Delta and increasing the infection rate in all countries (17). In Brazil, a combination of several factors, such as more transmissible VOCs (most notably Gamma and Omicron); limited application and engagement of nonpharmacological control measures; and a systematically weakened public health system, lamentably resulted in over 34 million cases and at least 680 thousand deaths up to November 2022 (2, 18–22).

Genomic surveillance in Brazil was limited during the beginning of the pandemic, but specific initiatives successfully detected and characterized the global introduction, community transmission, as well as the introduction and spread of VOCs (23–25). With the establishment of research networks, SARS-CoV-2 surveillance has improved substantially in the country, providing a precise understanding of transmission chains between states (26–29). But, up to October 2022, there were limited studies on intrastate SARS-CoV-2 transmission. Only Amazonas, Santa Catarina, Tocantins, Paraná, São Paulo, and Rio de Janeiro intrastate dynamics have been investigated (26, 29–33) as well as some reports from Rio Grande do Sul and Minas Gerais states focusing on specific lineage replacements (23, 34, 35). In Pernambuco state, we characterized multiple introductions and ongoing community transmission at the beginning of the pandemic (36). Although these studies provided crucial information to understand the SARS-CoV-2 dynamics in Brazil states or regions, SARS-CoV-2 lineage dynamics are expected to be region-specific due to the large differences between inter- and intrastate population sizes, population concentration, and human movement patterns in Brazil (26). In addition, nonpharmacological strategies to control SARS-CoV-2 were heterogeneously implemented across the country (18). Therefore, understanding the dynamics of SARS-CoV-2 within individual states taking into consideration the specific state characteristics is of paramount importance to understand the main determinants of virus spread at a fine-grained resolution.

We have sequenced 1,389 SARS-CoV-2 genomes from Pernambuco state in this retrospective study. Analyzing the new data set plus the available genomic sequences from the state, we investigate the COVID-19 pandemic in the state in a lineage transmission dynamics perspective from June 2020 to early August 2021. We describe the timing and spread dynamics of different infection waves in Pernambuco, considering geographical traffic data from federal and state highways as a proxy of human mobility within the state. Our results show that the rapid spread of both VOC and non-VOC lineages is driven by more populous urban centers located in the east and west sides of Pernambuco, toward the inner and less populated counties. The principal intrastate highway traffic mirrored this spread pattern and was likely a key component driving the SARS-CoV-2 lineage’s dissemination across the state.

## RESULTS AND DISCUSSION

### Genomic spreading of lineages of SARS-CoV-2 in the Pernambuco state

**Fig 1:**
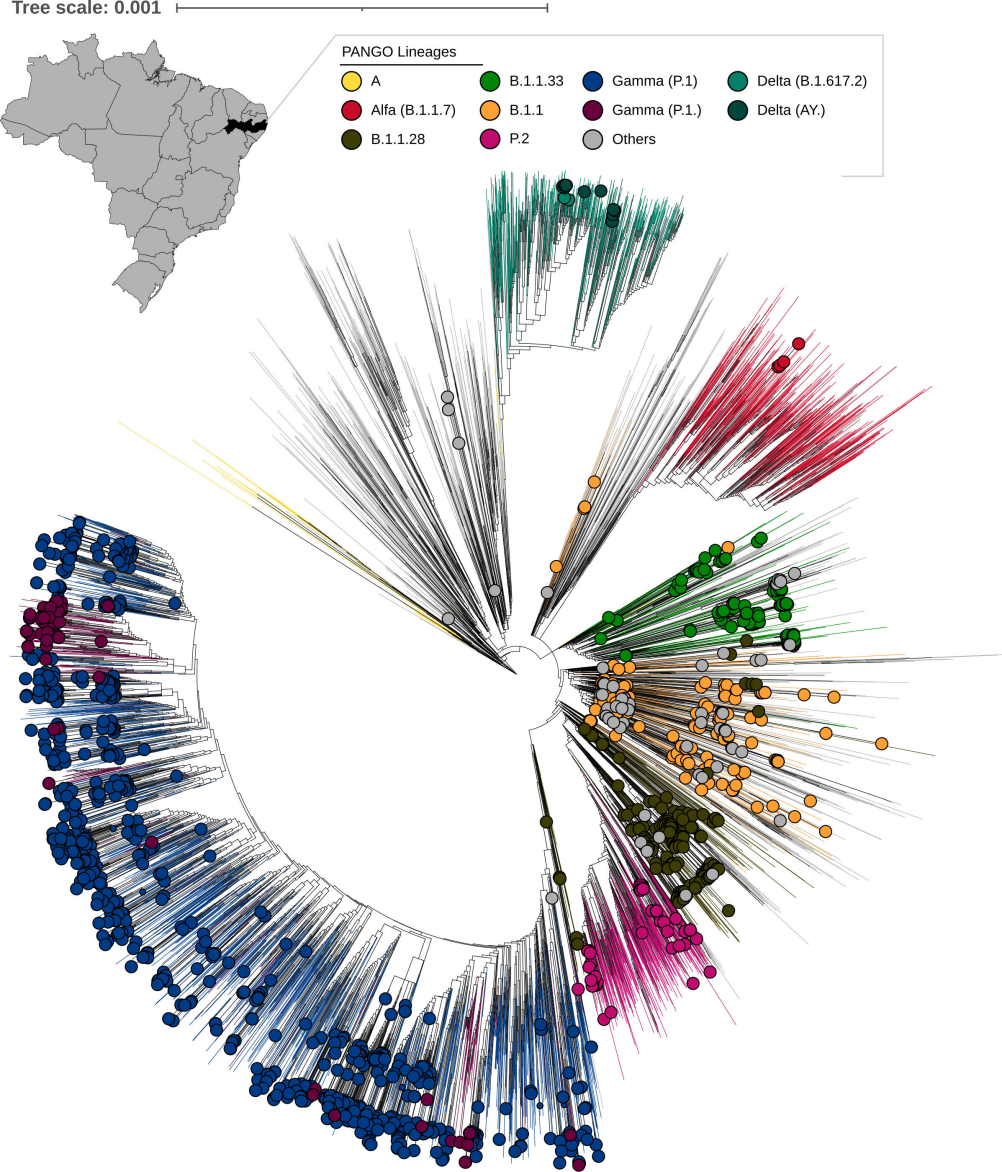
Brazilian map and federal states highlighting the Pernambuco state and maximum likelihood phylogenetic reconstruction of the SARS-CoV-2 global data set including 7,228 sequences; genomes generated from the Pernambuco state are marked with circles in the tip labels, and colors branches denote lineages.

**Fig 2:**
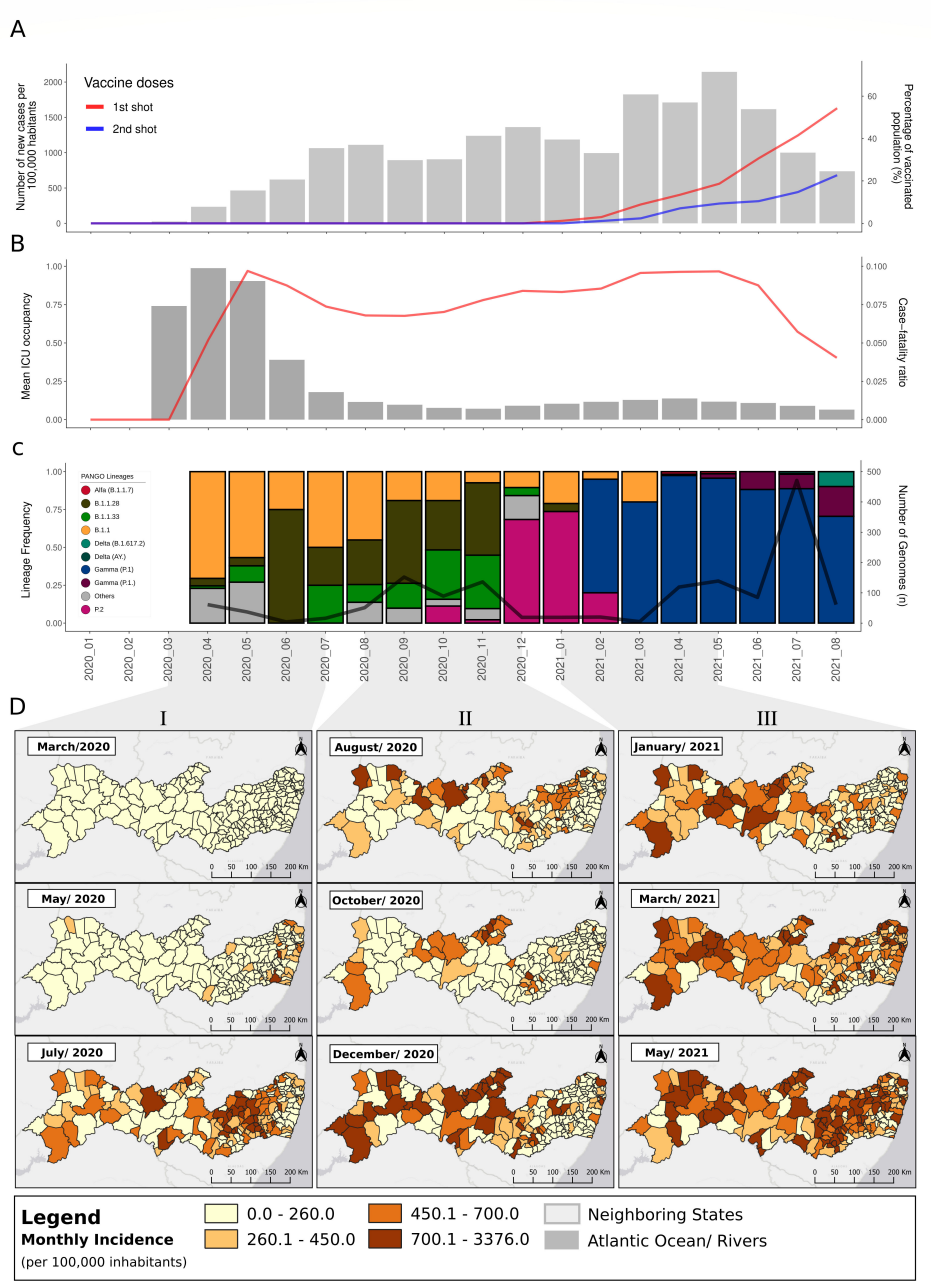
(**A**) Number of SARS-CoV-2 new cases per 100,000 habitants (gray bars) and percentage of Pernambuco population vaccinated based on the Pernambuco population (9,674,793) estimated in 2021 by the Instituto Brasileiro de Geografia e Estatística (IBGE), X axis: number of cases per 100,000 habitants, Y axis: From Jan 2020 to Aug 2021. ; (**B**) Case-fatality ratio (gray bars) and mean ICU occupancy (red line) in Pernambuco by period, X axis: mean ICU occupancy, Y axis: months Jan 2020 and Aug 2021; (**C**) SARS-CoV-2 lineage frequencies and number of genomes (black line) by period; (**D**) monthly incidence of SARS-CoV-2 infections across Pernambuco state.

We obtained 1,389 new genomes with an average coverage breadth and depth of 99.65 (st dev 1.57) and 487.27 (st dev 506.99), respectively, encompassing samples collected from 1st June 2020 to 9th August 2021 (see Table S1 at https://doi.org/10.6084/m9.figshare.22643194.v4). The first confirmed SARS-CoV-2 infections in Pernambuco, found in an elderly couple returning from Italy on 28 February, was reported in the second week of March (12 March 2020) in the 11th epidemiological week, 16 days after the first confirmed case in Brazil (25 February 2020) (37). Soon after the first case of SARS-CoV-2 infection in the state, we sequenced 101 genomes, from April to mid-May 2020, which were all classified in the B lineage representing multiple introductions of SARS-Cov-2 in the state (36). The present work is an update of the genomic surveillance comprising the sequencing and analysis of 1,389 new genomes from Pernambuco covering the emergence and spread of at least six epidemiologically important lineages: B1.1, B.1.1.33, B.1.1.28, P.2 (Zeta), P.1 (Gamma), and B.1.617.2 - AY.* (Delta) (see Fig. 1; Table S1 at https://doi.org/10.6084/m9.figshare.22643194.v4). The pandemic in Pernambuco state was characterized by two large case incidence and death peaks: one at the first semester of 2020 that stabilized during the second semester and a large peak at the first semester of 2021 (Fig. 2A); however, it is important to note that many cases were likely underreported during the beginning of the pandemic due to limited testing capacity. Genomes sequenced in this study covered all months, representing about 0.5% of all cases reported in the state.

Our analyses indicated the most prevalent lineage during the first infection wave (March–May 2020) at Pernambuco was B.1.1 detected from March to April 2020 (Fig. 2C). During October 2020, the first cases of the lineage P.2 were detected in Pernambuco, and at the time, P.2 was considered a variant of interest (VOI Zeta) due to specific spike mutations and increasing prevalence in Brazil (38, 39). Nevertheless, the B.1.1, B.1.1.33, and B.1.1.28 lineages remained the most prevalent lineages between October and November 2020. However, between December 2020 and January 2021, the P.2 lineage became the most prevalent lineage with only a moderate increase in case numbers and near stabilization of ICU bed occupancy (Fig. 2B). Then, the VOC Gamma (P1) arrived in the state in February 2021 and rapidly replaced all previous circulating lineages, becoming the most prevalent lineage from February until August 2021 (Fig. 2C). These findings are corroborated by the sequencing of the S gene in additional 135 independent SARS-CoV-2 samples (see Fig. S1 at https://doi.org/10.6084/m9.figshare.22643194.v4). During this period, case numbers increased, reaching a highest peak at that point of the pandemic. It is important to note that during the time frame, in which Zeta to Gamma lineages co-circulated and transitioned (January–February 2021), the increase in case numbers stabilized (Fig. 2A and C).

In January 2021, the vaccination campaign started in the state, but due to a slow rollout, it most likely had a limited impact on cases and deaths during the Gamma wave: as of August 2021, only 25% of the local population were fully vaccinated (two vaccine doses) (Fig. 2A). Therefore, the stabilization of ICU bed occupancy and deaths (March and May 2021) and its progressive decrease in case numbers after June 2021 are more likely explained by a composite effect of the immunological barrier acquired from natural infection of previous non-VOC lineages, by the widespread infection of the population by the Gamma lineage itself (40), and/or to the more intense adherence of the population to nonpharmacological intervention (NPI) during high health system pressure (18, 41). NPIs (multidisciplinary and multi-sectoral measures, social distancing and border control, and obligation of face masks in public environments) were unevenly implemented throughout Pernambuco and for short periods of time, but they probably contributed to the reduction of virus transmission in the state (42). Finally, another VOC, Delta, was introduced to the state in July 2021 and gradually replaced the dominant Gamma lineage without a major increase in case numbers and deaths (Fig. 2A through C), likely due to the previous immunity barrier of the population, and one dose of the vaccination reached to around 60% in August 2021. (Fig. 2C).

The analysis of the SARS-CoV-2 incidence through time and its association with lineage assignments in different Pernambuco counties revealed three distinct epidemic periods: **I:** March to July 2020 was characterized by a large increase in the incidence (incidence peak 398/100,000 inhabitants) mostly driven by the east largest metropolitan region of the state—the Recife Metropolitan Area (Fig. 2D). This period of the pandemics was characterized by a rapid increase in cases, reaching the peak in May 2020, which was dominated by three major cocirculating lineages B.1.1, B.1.1.28, and B.1.1.33. During the same month, there was clear evidence of interiorization in which the incidence increased in the west part of the state during June/July 2020, which was followed by extensive transmission of all dominating lineages covering the entire state. The months August and September were characterized by an overall decline in incidence (300–200 cases per 100,000 habitants) in the entire state (Fig. 2D). **II:** August to December 2020 was characterized by the emergence of the P.2 lineage in the east region of the state, which occurred concomitantly to a new increase in the incidence (incidence peak 458 cases by 100,00 inhabitants), which was more pronounced in the west and middle portions of the state . The increase in cases in the west and middle regions of the state was likely mainly driven by extensive community transmission of lineages B.1.1.28 and B.1.1.33 and P.2 (Fig. 2D). However, the low number of genomes obtained during this period may have limited the inferences of lineage prevalence. **III**: January to May 2021 was characterized by the arrival of the P.1 (Gamma) lineage that replaced all previous circulating lineages between February and March and drove the rapid increase in the incidence all over the state from March 2021 (Fig. 2D) (data set: https://doi.org/10.6084/m9.figshare.22643194.v4) (43). During the same period, we performed Sanger sequencing genotyping for a distinct set of samples. The findings corroborated the rapid P2 and B.1 lineage displacement by the Gamma (P.1) lineage between January and April 2021. Of note, in this subset of samples, the Gamma lineage represented 4.7% of the samples collected in January, 64% in February, 88% in March, and 100% in April (see Fig. S1 at https://doi.org/10.6084/m9.figshare.22643194.v4). The peak of infection cases was in May (incidence peak 769 cases per 100,000 inhabitants), followed by a continued decline in cases up to August 2021. In the same period, we also detected the emergence of the Delta lineage in the east part of the state (Fig. 2C).

### SARS-CoV-2 clades detected in Pernambuco state

**Fig 3:**
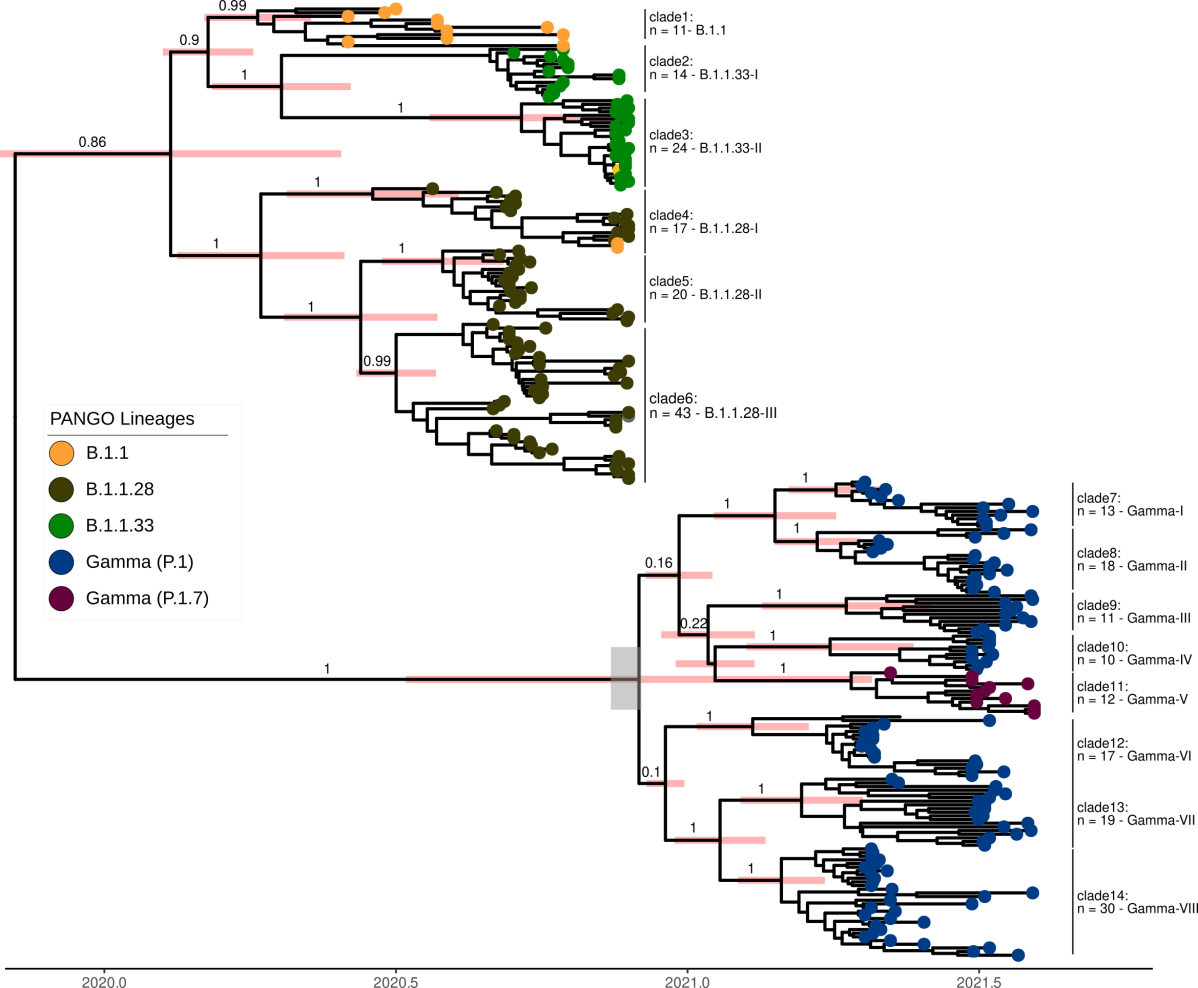
Temporal analysis of SARS-CoV-2 clades detected in the Pernambuco state. The number above the node shows the posterior probability support. The pink bars are the HPD 95% credible interval of the estimated tMRCA estimated on BEAST analysis. The gray rectangle represents the estimated origin of the Gamma lineage in the Amazonas state (32).

We detected fourteen SARS-CoV-2 monophyletic clades containing only Pernambuco samples until the first half of 2021 (Fig. 3). Our Bayesian analysis showed that the single B.1 clade showed the most ancient last common ancestor in late April to mid-May of 2020 (Table 1), which is in line with the emergence and predominance of this clade in the first weeks of SARS-CoV-2 transmission in the state, as observed from our previous study (36). Moreover, three different clades of B.1.1.28 cocirculated with two B.1.1.33 clades in the second semester of 2020—late June to late October—showing similar most recent common ancestor (tMRCA) dating (Table 1; Fig. 3). Lastly, we detected at least eight clades of Gamma (P.1) lineage in the state, which replaced all previous circulating lineages (Fig. 3). This pattern mirrors the P.1 (Gamma) replacements that occurred in other regions of Brazil. For instance, the B.1.1.28 was prevalent in the Amazonas state during the second semester of 2020; however, shortly after the emergence of the P.1-Gamma lineage, it was rapidly replaced by this VOC lineage (44). Likewise, the same was observed in other Brazilian states (45, 46). After the origin of the Gamma lineage in the Amazonas state in late November of 2020 (32), the subsequent transmission to other Brazilian states occurred mainly between December of 2020 and March of 2021 (47). Current studies estimate that the entry of the Gamma lineage in the northeast region varies from middle–late December of 2020 (47) to early January 2021 (26), which is consistent with the tMRCA of the Gamma lineage in the Pernambuco state (1st December 2020 [95% high posterior density (HPD): 7th November 2020–6th January 2021). Regarding the root ages of Gamma clades that seeded large transmission chains in the state, our analysis estimates that they date back between early February 2021 (clade 12, Table 1) and mid-April 2021 (Fig. 3; Table 1).

**TABLE 1:** tMRCA and HPD intervals of each identified clade in Fig. 3

Clade	Number of sequences	Lineages	tMRCA	95% HPD min.	95% HPD max.
1	11	B.1.1	2020–04-24	2020–03-26	2020–05-19
2	14	B.1.1.33	2020–09-09	2020–08-27	2020–09-13
3	24	B.1.1.33	2020–10-05	2020–09-08	2020–10-28
4	17	B.1.1.28	2020–07-18	2020–06-29	2020–07-25
5	20	B.1.1.28	2020–08-25	2020–08-12	2020–09-03
6	43	B.1.1.28	2020–07-21	2020–06-23	2020–08-13
7	13	Gamma	2021–04-17	2021–04-11	2021–04-19
8	18	Gamma	2021–04-17	2021–03-20	2021–04-26
9	11	Gamma	2021–05-16	2021–04-16	2021–06-12
10	10	Gamma	2021–04-26	2021–03-14	2021–06-02
11	12	Gamma	2021–05-02	2021–04-19	2021–05-07
12	17	Gamma	2021–03-12	2021–02-01	2021–04-19
13	19	Gamma	2021–04-03	2021–03-10	2021–04-24
14	30	Gamma	2021–03-17	2021–02-21	2021–04-05

### Inter- and intrastate terrestrial transportation dynamics

**Fig 4:**
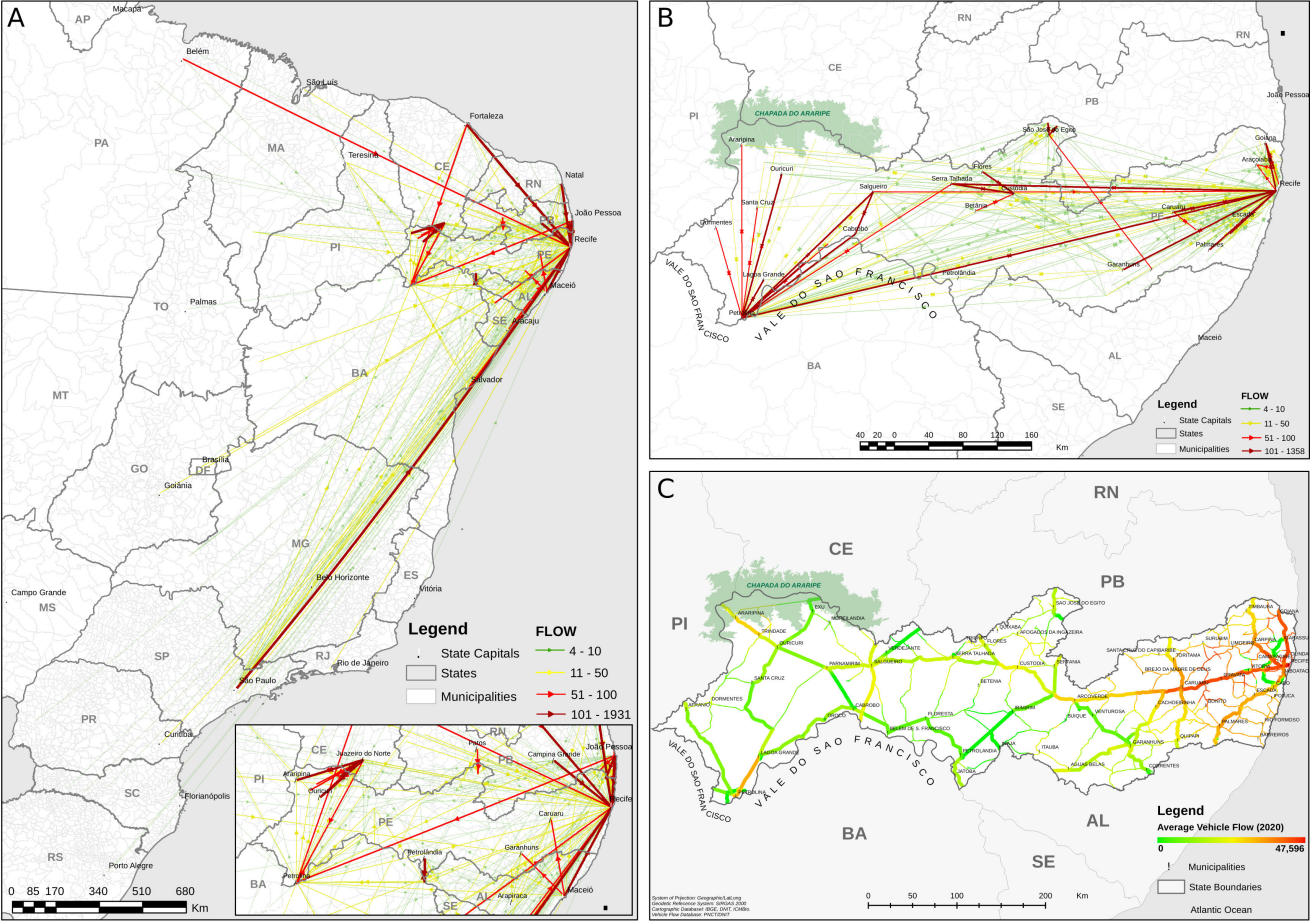
Origin and density of vehicle mobility in the state of Pernambuco. (**A**) shows the origin/destination intensity of vehicles departing from elsewhere in Brazil to the state of Pernambuco in 2018. (**B**) shows the destination from vehicles with both origin and destination within the state of Pernambuco. (**C**) shows the modeling of the average 2020 vehicle flux intensity on the roads according to the automated vehicle counters distributed throughout the region. Capital letters denotes Brazilian state acronyms as follows: AP: Amapá; PA: Pará; MA: Maranhão; PI: Piauí; CE: Ceará; RN: Rio Grande do Norte; TO: Tocantins; PB: Paraíba; PE: Pernambuco; BA: Bahia; MT: Mato Grosso; AL: Alagoas; SE: Sergipe; GO: Goiás; DF: Distrito Federal; MG: Minas Gerais; ES: Espírito Santo; MS: Mato Grosso do Sul; SP: São Paulo; RJ: Rio de Janeiro; PR: Paraná; SC: Santa Catarina; and RS: Rio Grande do Sul.

We detected not only a pronounced east–west dispersion pattern from the metropolitan region to the inner part of the state for all lineages but also a higher concentration of cases in the municipalities that border the states of Pernambuco on the north and south, indicating a spatial discontinuity with the east–west dispersion pattern (Fig. 2D and 4E zoomed section, Supplementary Videos and https://microreact.org/project/k9UFJmkrZHSx9RmFbDwZah-sars-cov-2-final-update-pernambuco). This finding may be related to the presence of main highways and trading of goods activities that connect these smaller municipalities to other regions from the neighboring states. Therefore, the movement of infected individuals from other states through means of ground transport may have contributed to further spread of the virus (Fig. 4A). It is important to note that we only used ground traffic data for intra-municipality mobility data in our analysis. Therefore, the results should be evaluated with caution as air transportation may also contribute substantially to respiratory virus transmission (48, 49). However, intrastate flight mobility in Pernambuco is expected to have a minor impact on human mobility compared to terrestrial transportation as there are only two large airports in Recife and Petrolina cities; hence, most of the human mobility within the state is expected to occur through highways.

At the interstate level, there is an overlap between the arrival and early circulation of SARS-CoV-2 lineages [B.1.1, B.1.1.28, Zeta (P.2), Gamma (P.1), and Delta (B.1.617.2)] in discontinued areas of the state and the interstate vehicle mobility flux. This finding can be evidenced on the western limit of Pernambuco (Chapada do Araripe and Vale do São Francisco regions), which borders the states of Ceará (Juazeiro do Norte) and Bahia (Paulo Afonso) and the central north part of the state that borders Paraíba (Patos). Those interstate regions are connected by important highway hubs (Fig. 4A and https://microreact.org/project/k9UFJmkrZHSx9RmFbDwZah-sars-cov-2-final-update-pernambuco). Despite that, vehicle mobility analysis showed that the Recife metropolitan area receives an intense flux of vehicles from othernNortheastern state capitals and from the cities of Belém in the north and São Paulo in the southeast (Fig. 4A). Based on that, the Recife metropolitan area (eastern Pernambuco) along with Araripina, Ouricuri, and Petrolina (Western Pernambuco) and Petrolândia (Central South Pernambuco) and São José do Egito (Central North Pernambuco) are likely the most important sink hubs of SARS-CoV-2 lineage arrival in the state and also the most important source hubs for intrastate transmission through terrestrial transportation.

Corroborating our data, studies conducted by different groups have led to the association of the dispersion of COVID-19 with transportations, such as buses, trains, and airplanes (50–52). A study conducted in Bahia, another Brazilian northeastern state, based on the spatial dispersion of COVID-19 also showed that the disease was distributed across the state through highways and airports (52). As observed in Recife, the capital of Pernambuco, and where the international airport is located, the highway network from the metropolitan region to the inner cities of the state likely had a predominant role in spreading the virus toward the country’s municipalities. While airports are well-known to act as a main hub of importation of new lineages from remote locations (53), the roads promote a spatially continuous spread at a smaller geographical scale.

While most of the mobility control measures focused on international hubs, such as airports and harbors, these results indicate that roads played a relevant role in the domestic spread of the virus (54). In Brazil, most of the anti-COVID-19 restrictive measures were autonomously designed by each one of the 27 state administrations without a centralized coordination (55). Therefore, it is possible that discrepant and asynchronous containment measures throughout the Brazilian states may have facilitated the SARS-CoV-2 lineage dispersion through highways at the regional (inter- municipality) level.

### Limitations of the study

We acknowledge some limitations of the present study. For example, although samples were obtained randomly at the geographical scale, the number of samples and genomes varied through time, which may have impacted the lineage spread and prevalence estimates across Pernambuco. Nevertheless, all tMRCA and lineage emergence and spread timing are in line with those of other publications that included Pernambuco samples, and the lineage dynamics agrees with findings across Brazilian states.

In addition, it is important to highlight that the amplification of specific genomic fragments (spanning the NTD and RBD spike protein) coupled with Sanger sequencing allows the distinction of some SARS-CoV-2 lineages, mainly VOC and non-VOC lineages. However, due to the limited number of lineage-defining mutations across the SARS-CoV-2 genomes as a whole, it is challenging to characterize sublineages and lineages that bear lineage-defining mutations outside the spike region. On the other hand, it is important to note that the data obtained with Sanger sequencing were incorporated in the current study only as a complementary information, eliminating the possible source of bias based on independently assessed samples through whole-genome sequencing and that our analysis of the VOC vs non-VOC pattern found with Sanger sequencing was in agreement with our findings based on whole-genome sequencing.

Lastly, although we explored the terrestrial intra- and interstate transportation, the data set available for this analysis did not allow us to perform a more fine-grained analysis throughout time. This is because only the yearly information was available (but not monthly). Therefore, intrayear traffic flow dynamics between municipalities, which very likely varied with the different nonpharmacological interventions implemented in Pernambuco, could not be evaluated. Aerial flux is also known to impact the SARS-CoV-2 lineage transmission dynamics, but most of the findings from the literature show a higher importance to explain intercontinental and interstate transmission. Within the Pernambuco state, there are only two major airports in the east and west regions, which probably resulted in a lower impact of aerial flux on intrastate dynamics when compared to terrestrial flux. Hence, the broad terrestrial traffic movement patterns depicted here should be interpreted with caution, and more studies are needed to assess and compare the importance of terrestrial vs aerial transportation on SARS-CoV-2 viral spread within the state.

### Conclusion

SARS-CoV-2 surveillance remains critical to understand how this virus evolves and spreads at different spatial and temporal scales. Here, through viral transmission chain reconstruction from SARS-CoV-2 genomic data and geospatial analysis, we characterized three main SARS-CoV-2 transmission waves in the Pernambuco state. Our findings corroborate the multiple lineage circulation dynamics of the first infection wave of the pandemic and the rapid replacement by the P.1 (Gamma) lineage, which dominated the second and larger epidemic wave that occurred in the first semester of 2021 and swiped through Brazil. Lastly, transport spatial analysis showed specific inter- and intrastate traffic flow patterns that can be leveraged to implement nonpharmacological actions to mitigate further SARS-CoV-2 and other high-impact respiratory virus transmission in both Pernambuco state and the northeast region of Brazil.

## MATERIAL AND METHODS

### Genomic sequencing

Nasopharyngeal samples were obtained from the Laboratório Central de Saúde Pública de Pernambuco (LACEN-PE) and the Núcleo de Pesquisa em Inovação Terapêutica - Suely Galdino (NUPIT-SG). RT-qPCR tests were performed using Kit Biomol OneStep COVID-19 (IBMP, Paraná, BR) and Kit Molecular SARS-CoV-2 (Bio-manguinhos, Rio de Janeiro, RJ, BR) according to the manufacturer. Samples with a cycle threshold (Ct) <25 were further processed for amplification and sequencing (56, 57). During the study period, we employed three different methodologies to generate cDNA and amplify the SARS-CoV-2 genome: short amplicon according to the ARTIC protocol (https://github.com/artic-network/artic-ncov2019) (56); long amplicon according to Resende, *et al*. 2020 (58); and inserting three sets of primers in the COVIDSeq protocol according to Naveca, *et al*. 2021 (59). Genome-wide amplicons were then processed for sequencing using Nextera XT (Illumina, San Diego, CA, USA) or COVIDSeq (Illumina, San Diego, CA, USA) library preparation protocols following the manufacturer’s instructions. Sequencing was performed using the MiSeq employing MiSeq Reagent kit V3 - paired-end 150 cycle flow cell. Ethical approval was obtained from CEP FIOCRUZ/IAM - CAAE 32333120.4.0000.5190.

### Genome assembly and annotation

A reference-guided genome assembly strategy was employed using ViralFlow v.0.0.6 (https://github.com/WallauBioinfo/ViralFlow) (60). Briefly, low-quality reads were trimmed in a sliding window mode of four bases with a mean Phred score threshold of 20, PCR primers and adapters were removed, and a minimum of 5 x coverage depth of bases with a Phred score quality equal to 30 were used for base-calling to the major consensus genome.

### Spike region genotyping analysis with Sanger sequencing

We performed a spike region genotyping of 135 additional SARS-CoV-2 RT-qPCR-positive samples between January and April 2021, using a mutation profile of the RBD region of the spike protein according to Bezerra 2021 *et al*. (61) with an updated primer set designed to flank the 22607–23446 region, (1 MS Fw 5′-TAACGCCACCAGATTTGCAT-3′ 2 MS Rv 5′-ACACGCCAAGTAGGAGTAAGT-3′) (dx.doi.org/10.17504/protocols.io.ewov1nxqkgr2/v2).

### Temporal and phylodynamic analysis

We recovered the full data set of 3,201,025 SARS-CoV-2 genomes from the GISAID database (https://www.gisaid.org/) on September 2, 2021. Subsequently, we performed a random subsampling with the Augur 6.3.0 (62) (https://docs.nextstrain.org/projects/augur/en/stable/index.html) pipeline obtaining 7,228 genome sequences (minimum 93% coverage) containing representative genomes from all continents and with focal context including 1,587 genomes from the Pernambuco state, where 1,490 were sequenced by the Aggeu Magalhães Institute at Fiocruz. The 7,228 genomes were aligned against the Wuhan-Hu-1 reference genome (GenBank NC_045512.2, GISAID EPI_ISL_402125) using MAFFT v7.47152, then the UTR regions were masked, and the phylogenetic tree was reconstructed with the neighbor joining method implemented in Augur. An additional phylogenetic tree was reconstructed with IQ-TREE2 (63) using the maximum likelihood approach with the model selected with ModelFinder (64) and SH-aLRT support values calculated from 1,000 replicates.

The temporal spreading pattern of the identified SARS-CoV-2 monophyletic clades was analyzed first using the maximum likelihood approach and microreact (https://microreact.org/project/k9UFJmkrZHSx9RmFbDwZah-sars-cov-2-final-update-pernambuco). To understand how many clades were circulating in the Pernambuco state, the initial phylogeny of 7,228 genomes was manually inspected, and the monophyletic clades with 10 or more genomes from Pernambuco showing clade support (aLRT) higher than 80 were recovered (GISAID accession number of genomes - doi:10.55876/gis8.230419ck (see Supplementary Material 1 at https://doi.org/10.6084/m9.figshare.22643194.v4). The resulting data set with 258 genomes was submitted to a Bayesian phylogenetic analysis with the Markov Chain Monte Carlo (MCMC) method implemented in the BEAST 1.10.4 (65) tool with BEAGLE library v.3 (66) to accelerate the computational analysis. The XML file was built with BEAUTI 1.10.4, using the collection date traits, the nonparametric Bayesian Skyline model (67), the evolutionary model under GTR + F + G4, and a strict clock with the initial value of the substitution rate of 8 × 10^−4^ substitutions/site/year and continuous-time Markov chain (CTMC) rate reference prior (68). Three runs of 200 million generations were independently performed, the trees were combined applying a burn-in of 25%, and the convergence of analysis was evaluated with TRACER v1.7 (69) to ensure the effective sample size (ESS) was higher than 200. The maximum clade credibility tree was summarized with TreeAnotator 1.10.4, and the consensus tree was plotted with ggtree (70). To assess the age root of each clade, a secondary Bayesian analysis was performed using data sets of highly supported clades (posterior probability ≥0.99) selected in the previous analysis; each data set was submitted to the same strategy as the first Bayesian analysis (XML files available on see Supplementary Material 2 at https://doi.org/10.6084/m9.figshare.22643194.v4).

### Epidemiological data

Data of SARS-CoV-2 lab-confirmed (RT-qPCR) cases and deaths from Brazil and Pernambuco state were recovered from the Brazilian Ministry of Health coronavirus database (https://covid.saude.gov.br/). We collected the number of cases and deaths per day and per epidemiological week since the first case reported in the state (March 12th to September 2nd) (2, 71). The data were parsed using dplyr (72) and tidyverse R packages, and plots were performed using the ggplot2 (73) package of the R statistical language (https://www.r-project.org/).

### Monthly maps of SARS-CoV-2 infection in the state of Pernambuco

We calculated the monthly incidence using the number of cases of each municipality, divided by the estimated population in the year 2020, and multiplied by 100,000 inhabitants. We collected data regarding SARS-CoV-2 cases from the “Covid 19 in Data” platform available on the website of the Planning Department of the State of Pernambuco, on https://covid.saude.gov.br/. For the map construction, the cartographic base of the municipalities of Pernambuco was collected, in shapefile format, geographic projection system (latitude/longitude), and geodetic reference system SIRGAS 2000, available on the IBGE (Brazilian Institute for Geography and Statistics) website. Subsequently, to visualize the incidence of SARS-CoV-2 infection in the state of Pernambuco, thematic maps categorized into quartiles were generated.

### Vehicle mobility flow maps in Brazil and Pernambuco

We generated maps of vehicle mobility flow, including all sorts of vehicles such as cars, buses, and trucks that were interviewed while trafficking in the roads during 2018 to identify displacement patterns considering the city of origin and destination. The database containing this information was made available through the Origin-Destination Survey of the PNCT (National Traffic Count Plan) by the DNIT (National Department of Infrastructure and Transport) through the link https://servicos.dnit.gov.br/dadospnct/PesquisaOD/BaseDeDados. We generated a new database to carry out the georeferencing analysis. We considered the following variables: municipality of origin and destination with geographic coordinates and flow density. We used the MMQGIS (Hub Lines/Distance) package of the QGIS 3.10 to elaborate the flow maps.

### Highway traffic map in Pernambuco

For spatial modeling of the flow density of vehicles on the highways of the state of Pernambuco, we used a database containing traffic counting information collected through electronic counters positioned at specific points on the highways of Pernambuco. We recovered this data set from the National Traffic Count Plan (PNCT) of the National Department of Infrastructure and Transport (DNIT) from http://servicos.dnit.gov.br/dadospnct/ContagemContinua. There are currently 22 electronic counting posts spread across the state that capture the number of crossing vehicles (cars, buses, trucks etc.) through time. A second database was generated with the bidirectional average flux for 2020 to carry out the georeferencing of the intensity of the flow of vehicles, considering the information referring to geographic coordinates and quantity of vehicles from each monitoring station as representative for each section of the road network. We also collected from the DNIT the digital cartographic database of the state’s highways, as well as the cartographic database of the municipalities of Pernambuco from the IBGE website. The database with vehicle traffic information was later added to the shapefile file of highways. To visualize the intensity of the flow of vehicles on the highways of Pernambuco, we used the ordinary kriging method (74). The maps were generated using ArcGIS 10.1 software.
